# Undeca­europium hexa­zinc dodeca­arsenide

**DOI:** 10.1107/S1600536810006938

**Published:** 2010-02-27

**Authors:** Bayrammurad Saparov, Svilen Bobev

**Affiliations:** a Department of Chemistry and Biochemistry, University of Delaware, Newark, DE 19716, USA

## Abstract

The title compound, Eu_11_Zn_6_As_12_, crystallizes with the Sr_11_Cd_6_Sb_12_ structure type (Pearson’s symbol *mC*58). The complex monoclinic structure of the first arsenide to form with this type features chains made of corner-sharing ZnAs_4_ tetra­hedra, separated by Eu atoms. There are a total of 15 unique positions in the asymmetric unit. Except for one Eu atom with site symmetry 2/*m*, all atoms are located on mirror planes. An usual aspect of the structure are some Zn—As distances, which are much longer than the sum of the covalent radii, indicating weaker inter­actions.

## Related literature

The growing inter­est in ternary pnictides of alkaline- and rare-earth metals with group 12 metals has feen fueled by the recent discovery of superconductivity (Rotter *et al.*, 2008 ▶). Such compounds have also been investigated because of their promising behaviour as materials with high thermoelectric conversion efficiency (Snyder & Toberer, 2008 ▶). Our own exploratory studies revealed a wealth of new compounds with diverse crystal structures, including Ca_2_CdSb_2_ and Yb_2_CdSb_2_ (Xia & Bobev, 2007*a* ▶), *A*_9_Cd_4+_ *_x_Pn*_9_ and *A*_9_Zn_4+_ *_x_Pn*_9_ (*A *= Ca, Sr, Eu, Yb; *Pn *= Sb, Bi) (Xia & Bobev, 2007*b* ▶), *A*_11_Cd_6_Sb_12_ (*A *= Sr, Ba, Eu) and Eu_11_Zn_6_Sb_12_ (Park & Kim, 2004 ▶; Xia & Bobev, 2008*b* ▶; Saparov *et al.*, 2008*a* ▶), *A*_21_Cd_4_ *Pn*_18_ (*A *= Sr, Ba, Eu; *Pn *= Sb, Bi) (Xia & Bobev, 2008*a* ▶), Ba_3_Cd_2_Sb_4_ (Saparov *et al.*, 2008*b* ▶), Ba_2_Cd_2_ *Pn*_3_ (*Pn *= As, Sb) (Saparov *et al.*, 2010 ▶). The title compound is the As-analog of Eu_11_Zn_6_Sb_12_ (Saparov *et al.*, 2008*a* ▶). For covalent radii, see: Pauling (1960 ▶).

## Experimental

### 

#### Crystal data

Eu_11_Zn_6_As_12_
*M* *_r_* = 2962.82
Monoclinic, 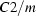

*a* = 30.310 (8) Å
*b* = 4.3318 (11) Å
*c* = 11.774 (3) Å
β = 109.746 (4)°
*V* = 1455.0 (7) Å^3^
*Z* = 2
Mo *K*α radiation
μ = 41.68 mm^−1^
*T* = 200 K
0.07 × 0.05 × 0.05 mm

#### Data collection

Bruker SMART APEX diffractometer
Absorption correction: multi-scan (*SADABS*; Bruker, 2002 ▶) *T*_min_ = 0.161, *T*_max_ = 0.256
7178 measured reflections
1796 independent reflections
1498 reflections with *I* > 2σ(*I*)
*R*_int_ = 0.042

#### Refinement

*R*[*F* ^2^ > 2σ(*F* ^2^)] = 0.028
*wR*(*F* ^2^) = 0.062
*S* = 1.01
1796 reflections
90 parameters
Δρ_max_ = 1.70 e Å^−3^
Δρ_min_ = −1.74 e Å^−3^

### 

Data collection: *SMART* (Bruker, 2002 ▶); cell refinement: *SAINT* (Bruker, 2002 ▶); data reduction: *SAINT*; program(s) used to solve structure: *SHELXTL* (Sheldrick, 2008 ▶); program(s) used to refine structure: *SHELXTL*; molecular graphics: *XP* in *SHELXTL*; software used to prepare material for publication: *SHELXTL*.

## Supplementary Material

Crystal structure: contains datablocks global, I. DOI: 10.1107/S1600536810006938/wm2307sup1.cif

Structure factors: contains datablocks I. DOI: 10.1107/S1600536810006938/wm2307Isup2.hkl

Additional supplementary materials:  crystallographic information; 3D view; checkCIF report

## Figures and Tables

**Fig. 1.**
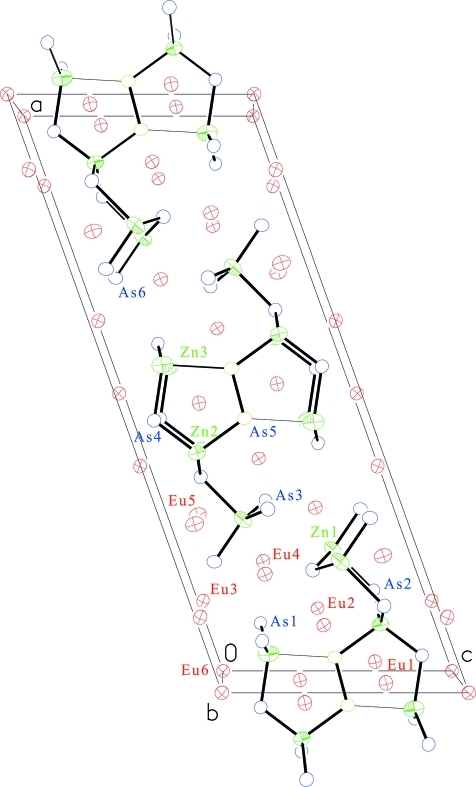
A plot of the Eu11Zn6As12 structure viewed down the b-axis. Displacement ellipsoids are drawn at the 95% probability level. Color key: Eu - red, Zn - green, All As atoms, excluding As5 - blue. As5, which form dimers are shown in yellow. The long As5—Zn3 bonds are depicted as thiner solid lines. The unit cell is outlined.

## supplementary crystallographic information

### Comment

The structure of Eu_11_Zn_6_As_12_ projected along *b*-axis is shown in Figure 1. The asymmetric unit is composed of 6 Eu, 3 Zn and 6 As atoms, all in special positions (Wyckoff position for Eu6 is 2*a*, for all others 4*i*).

The anionic substructure is made of Zn-centered ZnAs_4_ tetrahedra that share common corners to form chains. The terminal As atoms of two chains are close together, so that they form a covalent bond. This can be inferred from the resulting As5—As5 distance at 2.457 (3) Å, which is on par with the As—As separation in elemental As (2.517 Å). All other interatomic distances fall within the expected range, excluding the Zn3—As5 distance at 3.288 Å. The latter is too long - more than 30% longer than the sum of the Pauling's covalent radii (Pauling, 1960) - to be considered a simple 2-center-2-electron bond. Analogously longer than normal Cd3—Sb5 and Zn3—Sb5 distances have been reported in Eu_11_Cd_6_Sb_12_ and Eu_11_Zn_6_Sb_12_ (Saparov *et al.*, 2008a). We refer to the theoretical studies on Sr_11_Cd_6_Sb_12_ and Ba_11_Cd_6_Sb_12_ (Xia & Bobev, 2008b) for a more detailed discussion of the bonding interactions in this structure type.

*d*-metal centered tetrahedra of the pnicogen elements are recurring motifs in the structural chemistry of such solid-state compounds, as evidenced by a number of reports (Rotter *et al.*, 2008; Snyder & Toberer, 2008; Xia & Bobev, 2008a; Saparov *et al.*, 2008b; Saparov *et al.*, 2010). Sr_11_Cd_6_Sb_12_, the first structurally characterized phase with this monoclinic structure, was synthesized from a high temperature reaction of elements using Sn as metal flux (Park & Kim, 2004). In this report, the crystal structure was described as being composed of "double pentagonal tubes". A slightly different description of the structure was given in the light of the very long Cd3—Sb5 bond in Ba_11_Cd_6_Sb_12_ (Xia & Bobev, 2008b). Therein, the authors performed comprehensive electronic structure calculations aimed at full understanding of the bonding in Sr_11_Cd_6_Sb_12_ and Ba_11_Cd_6_Sb_12_. From these computational results, and from earlier results pertaining to related materials such as Yb_2_CdSb_2_ (Xia & Bobev, 2007*a*), *A*_9_Cd_4+_*_x_Pn*_9_ and *A*_9_Zn_4+_*_x_Pn*_9_ (*A*=Ca, Sr, Eu, Yb; *Pn*= Sb, Bi) (Xia & Bobev, 2007*b*), it can be expected that the Eu cations in Eu_11_Zn_6_As_12_ will be divalent, and the spins of the Eu's 7 unpaired electrons may couple magnetically at low temperatures.  We were unable to experimentally confirm this conjecture because the title compound was not isolated as a pure phase, but magnetic property measurements on the isotypic europium antimonides Eu_11_Cd_6_Sb_12_ and Eu_11_Zn_6_Sb_12_ (Saparov *et al.*, 2008a) confirmed Eu^2+^ cations (4*f*^7^ state). These measurements also suggested antiferromagnetic ordering in Eu_11_Cd_6_Sb_12_ below *T*_N_=7.5 K. The temperature dependent electrical resistivity measurements carried out on a single crystal of Eu_11_Cd_6_Sb_12_ suggested poorly metallic behavior, as expected from band structure calculations performed for Sr_11_Cd_6_Sb_12_ and Ba_11_Cd_6_Sb_12_ (Xia & Bobev, 2008b).

### Experimental

The starting materials, Eu, Zn, As, and Pb, with stated purity greater than 99.9%, were purchased from Alfa or Aldrich, and used as received. Elements were loaded into an alumina crucible in a Eu:Zn:As:Pb=2:1:2:10 molar ratio inside an argon-filled glove-box. The alumina crucible was then sealed under vacuum in a silica tube. The reaction mixture was heated to 1223 K, kept at this temperature for 20 hours, and then slowly cooled to 723 K at a rate of 3 K/hour. Finally, the Pb-flux was removed by centrifugation at this temperature. Together with irregular-shaped crystals with hitherto unknown structure, black needle shaped crystals of Eu_11_Zn_6_As_12_ were also obtained.

### Refinement

The collected data were successfully refined using the coordinates of Eu_11_Zn_6_Sb_12_ (Saparov *et al.*, 2008a) as a starting model. The maximum peak and deepest hole are located 0.97 Å away from Eu6 and 1.47 Å away from Zn1, respectively.

### Crystal data

**Table:**

Eu_11_Zn_6_As_12_	*F*(000) = 2538
*M**_r_* = 2962.82	*D*_x_ = 6.763 Mg m^−^^3^
Monoclinic, *C*2/*m*	Mo *K*α radiation, λ = 0.71073 Å
Hall symbol: -C 2y	Cell parameters from 1796 reflections
*a* = 30.310 (8) Å	θ = 1.4–27.1°
*b* = 4.3318 (11) Å	µ = 41.68 mm^−^^1^
*c* = 11.774 (3) Å	*T* = 200 K
β = 109.746 (4)°	Needle, black
*V* = 1455.0 (7) Å^3^	0.07 × 0.05 × 0.05 mm
*Z* = 2	

### Data collection

**Table:**

Bruker SMART APEX diffractometer	1796 independent reflections
Radiation source: fine-focus sealed tube	1498 reflections with *I* > 2σ(*I*)
graphite	*R*_int_ = 0.042
ω scans	θ_max_ = 27.1°, θ_min_ = 1.4°
Absorption correction: multi-scan (*SADABS*; Bruker, 2002)	*h* = −38→38
*T*_min_ = 0.161, *T*_max_ = 0.256	*k* = −5→5
7178 measured reflections	*l* = −15→15

### Refinement

**Table:**

Refinement on *F*^2^	Primary atom site location: structure-invariant direct methods
Least-squares matrix: full	Secondary atom site location: difference Fourier map
*R*[*F*^2^ > 2σ(*F*^2^)] = 0.028	*w* = 1/[σ^2^(*F*_o_^2^) + (0.0242*P*)^2^ + 16.9522*P*] where *P* = (*F*_o_^2^ + 2*F*_c_^2^)/3
*wR*(*F*^2^) = 0.062	(Δ/σ)_max_ < 0.001
*S* = 1.01	Δρ_max_ = 1.70 e Å^−^^3^
1796 reflections	Δρ_min_ = −1.74 e Å^−^^3^
90 parameters	Extinction correction: *SHELXTL* (Sheldrick, 2008), Fc^*^=kFc[1+0.001xFc^2^λ^3^/sin(2θ)]^-1/4^
0 restraints	Extinction coefficient: 0.000263 (17)

### Special details

**Table:**

Experimental. Selected in the glove box, crystals were put in a Paratone N oil and cut to the desired dimensions. The chosen crystal was mounted on a tip of a glass fiber and quickly transferred onto the goniometer. The crystal was kept under a cold nitrogen stream to protect from the ambient air and moisture.Data collection is performed with four batch runs at φ = 0.00 ° (600 frames), at φ = 90.00 ° (600 frames), at φ = 180.00 ° (600 frames), and at φ = 270.00 (600 frames). Frame width = 0.30 \& in ω. Data are merged and treated with multi-scan absorption corrections.
Geometry. All esds (except the esd in the dihedral angle between two l.s. planes) are estimated using the full covariance matrix. The cell esds are taken into account individually in the estimation of esds in distances, angles and torsion angles; correlations between esds in cell parameters are only used when they are defined by crystal symmetry. An approximate (isotropic) treatment of cell esds is used for estimating esds involving l.s. planes.
Refinement. Refinement of *F*^2^ against ALL reflections. The weighted *R*-factor wR and goodness of fit *S* are based on *F*^2^, conventional *R*-factors *R* are based on *F*, with *F* set to zero for negative *F*^2^. The threshold expression of *F*^2^ > σ(*F*^2^) is used only for calculating *R*-factors(gt) etc. and is not relevant to the choice of reflections for refinement. *R*-factors based on *F*^2^ are statistically about twice as large as those based on *F*, and *R*- factors based on ALL data will be even larger.

### Fractional atomic coordinates and isotropic or equivalent isotropic displacement parameters (Å^2^)

**Table:**

	*x*	*y*	*z*	*U*_iso_*/*U*_eq_	
Eu1	0.01672 (2)	0.0000	0.67859 (6)	0.01461 (16)	
Eu2	0.11288 (2)	0.0000	0.51100 (6)	0.01520 (16)	
Eu3	0.12610 (2)	0.0000	0.02177 (6)	0.01671 (17)	
Eu4	0.19815 (2)	0.0000	0.34699 (6)	0.01708 (16)	
Eu5	0.28179 (2)	0.0000	0.13448 (6)	0.01841 (17)	
Eu6	0.0000	0.0000	0.0000	0.0151 (2)	
As1	0.08620 (4)	0.0000	0.23794 (11)	0.0139 (3)	
As2	0.14491 (4)	0.0000	0.78289 (12)	0.0141 (3)	
As3	0.30702 (5)	0.0000	0.45283 (12)	0.0147 (3)	
As4	0.45498 (4)	0.0000	0.12128 (12)	0.0137 (3)	
As5	0.45839 (4)	0.0000	0.49049 (12)	0.0139 (3)	
As6	0.70775 (4)	0.0000	0.14605 (12)	0.0150 (3)	
Zn1	0.21901 (6)	0.0000	0.66996 (14)	0.0204 (3)	
Zn2	0.40088 (5)	0.0000	0.24844 (14)	0.0175 (3)	
Zn3	0.54783 (6)	0.0000	0.23600 (16)	0.0226 (4)	

### Atomic displacement parameters (Å^2^)

**Table:**

	*U*^11^	*U*^22^	*U*^33^	*U*^12^	*U*^13^	*U*^23^
Eu1	0.0163 (3)	0.0135 (3)	0.0139 (3)	0.000	0.0050 (3)	0.000
Eu2	0.0129 (3)	0.0186 (3)	0.0146 (3)	0.000	0.0053 (3)	0.000
Eu3	0.0155 (3)	0.0209 (3)	0.0146 (4)	0.000	0.0063 (3)	0.000
Eu4	0.0142 (3)	0.0209 (3)	0.0164 (4)	0.000	0.0056 (3)	0.000
Eu5	0.0159 (3)	0.0153 (3)	0.0249 (4)	0.000	0.0080 (3)	0.000
Eu6	0.0138 (5)	0.0176 (4)	0.0142 (5)	0.000	0.0050 (4)	0.000
As1	0.0137 (6)	0.0153 (6)	0.0129 (7)	0.000	0.0048 (5)	0.000
As2	0.0145 (6)	0.0131 (6)	0.0149 (7)	0.000	0.0055 (5)	0.000
As3	0.0153 (7)	0.0135 (6)	0.0149 (7)	0.000	0.0047 (5)	0.000
As4	0.0136 (6)	0.0146 (6)	0.0135 (7)	0.000	0.0052 (5)	0.000
As5	0.0116 (6)	0.0140 (6)	0.0163 (7)	0.000	0.0050 (5)	0.000
As6	0.0129 (6)	0.0148 (6)	0.0164 (7)	0.000	0.0040 (5)	0.000
Zn1	0.0208 (8)	0.0175 (7)	0.0177 (8)	0.000	−0.0005 (7)	0.000
Zn2	0.0146 (7)	0.0165 (7)	0.0218 (8)	0.000	0.0066 (6)	0.000
Zn3	0.0197 (8)	0.0145 (7)	0.0330 (10)	0.000	0.0080 (7)	0.000

### Geometric parameters (Å, °)

**Table:**

Eu1—As4^i^	3.1006 (12)	Eu6—Zn3^viii^	3.4339 (15)
Eu1—As4^ii^	3.1006 (12)	Eu6—Zn3^iii^	3.4339 (15)
Eu1—As5^iii^	3.1742 (12)	Eu6—Eu3^xi^	3.7434 (12)
Eu1—As5^iv^	3.1742 (12)	As1—Zn3^iii^	2.4548 (11)
Eu1—Zn2^i^	3.1965 (13)	As1—Zn3^iv^	2.4548 (11)
Eu1—Zn2^ii^	3.1965 (13)	As1—Eu1^v^	3.5733 (16)
Eu1—As5^ii^	3.1997 (12)	As2—Zn2^ii^	2.5313 (12)
Eu1—As5^i^	3.1997 (12)	As2—Zn2^i^	2.5313 (12)
Eu1—Zn3^ii^	3.2954 (14)	As2—Zn1	2.971 (2)
Eu1—Zn3^i^	3.2954 (14)	As2—Eu5^i^	3.0187 (11)
Eu1—As1^v^	3.5732 (16)	As2—Eu5^ii^	3.0187 (11)
Eu1—As2	3.6580 (17)	As2—Eu3^xii^	3.0526 (16)
Eu2—As2	3.0150 (17)	As3—Zn1^ii^	2.5754 (12)
Eu2—As1	3.0386 (16)	As3—Zn1^i^	2.5754 (12)
Eu2—As5^ii^	3.0548 (11)	As3—Eu2^i^	3.1731 (12)
Eu2—As5^i^	3.0548 (11)	As3—Eu2^ii^	3.1731 (12)
Eu2—Zn1	3.1276 (18)	As3—Eu4^i^	3.2434 (12)
Eu2—As3^i^	3.1730 (12)	As3—Eu4^ii^	3.2434 (12)
Eu2—As3^ii^	3.1730 (12)	As4—Zn2	2.567 (2)
Eu2—Zn2^i^	3.6993 (15)	As4—Zn3	2.679 (2)
Eu2—Zn2^ii^	3.6993 (15)	As4—Eu1^i^	3.1006 (12)
Eu2—Eu4	3.7123 (11)	As4—Eu1^ii^	3.1006 (12)
Eu2—Eu1^v^	3.8063 (13)	As4—Eu6^xiii^	3.1477 (10)
Eu3—As2^vi^	3.0526 (16)	As4—Eu6^xiv^	3.1477 (10)
Eu3—As1	3.1656 (15)	As4—Eu3^vii^	3.2820 (12)
Eu3—As6^iv^	3.2419 (12)	As4—Eu3^viii^	3.2820 (12)
Eu3—As6^iii^	3.2419 (12)	As5—As5^xv^	2.456 (3)
Eu3—As4^vii^	3.2819 (12)	As5—Zn2	2.794 (2)
Eu3—As4^viii^	3.2819 (12)	As5—Eu2^ii^	3.0548 (11)
Eu3—Zn2^vii^	3.7045 (16)	As5—Eu2^i^	3.0548 (11)
Eu3—Zn2^viii^	3.7045 (16)	As5—Eu1^xiv^	3.1743 (12)
Eu3—Eu4	3.7122 (13)	As5—Eu1^xiii^	3.1743 (12)
Eu3—Eu6	3.7434 (12)	As5—Eu1^ii^	3.1996 (12)
Eu3—Eu1^vi^	4.2739 (13)	As5—Eu1^i^	3.1996 (12)
Eu3—Eu3^ix^	4.3318 (11)	As6—Zn1^xv^	2.524 (2)
Eu4—As3	3.1086 (16)	As6—Eu5^xiv^	3.1547 (12)
Eu4—As1	3.1966 (16)	As6—Eu5^xiii^	3.1547 (12)
Eu4—As3^i^	3.2435 (12)	As6—Eu3^xiii^	3.2418 (12)
Eu4—As3^ii^	3.2435 (12)	As6—Eu3^xiv^	3.2418 (12)
Eu4—As6^iii^	3.2922 (13)	As6—Eu4^xiv^	3.2922 (13)
Eu4—As6^iv^	3.2922 (13)	As6—Eu4^xiii^	3.2922 (13)
Eu4—Zn1^i^	3.3727 (15)	As6—Eu5^x^	3.4229 (17)
Eu4—Zn1^ii^	3.3727 (15)	Zn1—As6^xv^	2.524 (2)
Eu4—Zn1	3.638 (2)	Zn1—As3^ii^	2.5754 (12)
Eu4—Eu5	4.1199 (12)	Zn1—As3^i^	2.5754 (12)
Eu5—As2^i^	3.0188 (11)	Zn1—Eu5^i^	3.1671 (14)
Eu5—As2^ii^	3.0188 (11)	Zn1—Eu5^ii^	3.1671 (14)
Eu5—As6^iii^	3.1546 (12)	Zn1—Eu4^i^	3.3727 (15)
Eu5—As6^iv^	3.1546 (12)	Zn1—Eu4^ii^	3.3727 (15)
Eu5—Zn1^i^	3.1672 (14)	Zn2—As2^ii^	2.5313 (12)
Eu5—Zn1^ii^	3.1672 (14)	Zn2—As2^i^	2.5313 (12)
Eu5—Zn2	3.3995 (19)	Zn2—Eu1^i^	3.1964 (13)
Eu5—As6^x^	3.4230 (17)	Zn2—Eu1^ii^	3.1964 (13)
Eu5—As3	3.5633 (18)	Zn2—Eu2^i^	3.6993 (15)
Eu5—Eu5^vii^	3.7827 (13)	Zn2—Eu2^ii^	3.6993 (15)
Eu5—Eu5^viii^	3.7827 (13)	Zn2—Eu3^vii^	3.7045 (16)
Eu6—As1^xi^	3.1191 (14)	Zn2—Eu3^viii^	3.7045 (16)
Eu6—As1	3.1191 (14)	Zn3—As1^xiv^	2.4548 (11)
Eu6—As4^vii^	3.1478 (10)	Zn3—As1^xiii^	2.4548 (11)
Eu6—As4^iv^	3.1478 (10)	Zn3—Eu1^ii^	3.2954 (14)
Eu6—As4^viii^	3.1478 (10)	Zn3—Eu1^i^	3.2954 (14)
Eu6—As4^iii^	3.1478 (10)	Zn3—Eu6^xiii^	3.4339 (15)
Eu6—Zn3^vii^	3.4339 (15)	Zn3—Eu6^xiv^	3.4339 (15)
Eu6—Zn3^iv^	3.4339 (15)		
			
As4^i^—Eu1—As4^ii^	88.62 (4)	As4^iv^—Eu6—As4^viii^	93.04 (4)
As4^i^—Eu1—As5^iii^	162.32 (4)	As1^xi^—Eu6—As4^iii^	92.72 (3)
As4^ii^—Eu1—As5^iii^	89.97 (3)	As1—Eu6—As4^iii^	87.28 (3)
As4^i^—Eu1—As5^iv^	89.97 (3)	As4^vii^—Eu6—As4^iii^	93.04 (4)
As4^ii^—Eu1—As5^iv^	162.32 (4)	As4^iv^—Eu6—As4^iii^	86.96 (4)
As5^iii^—Eu1—As5^iv^	86.05 (4)	As4^viii^—Eu6—As4^iii^	180.00 (5)
As4^i^—Eu1—Zn2^i^	48.09 (4)	As1^xi^—Eu6—Zn3^vii^	43.67 (2)
As4^ii^—Eu1—Zn2^i^	106.18 (4)	As1—Eu6—Zn3^vii^	136.33 (2)
As5^iii^—Eu1—Zn2^i^	148.25 (4)	As4^vii^—Eu6—Zn3^vii^	47.79 (4)
As5^iv^—Eu1—Zn2^i^	85.74 (3)	As4^iv^—Eu6—Zn3^vii^	132.21 (4)
As4^i^—Eu1—Zn2^ii^	106.18 (4)	As4^viii^—Eu6—Zn3^vii^	101.31 (4)
As4^ii^—Eu1—Zn2^ii^	48.09 (4)	As4^iii^—Eu6—Zn3^vii^	78.69 (4)
As5^iii^—Eu1—Zn2^ii^	85.74 (3)	As1^xi^—Eu6—Zn3^iv^	136.33 (2)
As5^iv^—Eu1—Zn2^ii^	148.25 (4)	As1—Eu6—Zn3^iv^	43.67 (2)
Zn2^i^—Eu1—Zn2^ii^	85.31 (4)	As4^vii^—Eu6—Zn3^iv^	132.21 (4)
As4^i^—Eu1—As5^ii^	151.99 (4)	As4^iv^—Eu6—Zn3^iv^	47.79 (4)
As4^ii^—Eu1—As5^ii^	86.40 (3)	As4^viii^—Eu6—Zn3^iv^	78.69 (4)
As5^iii^—Eu1—As5^ii^	45.32 (4)	As4^iii^—Eu6—Zn3^iv^	101.31 (4)
As5^iv^—Eu1—As5^ii^	102.75 (3)	Zn3^vii^—Eu6—Zn3^iv^	180.00 (5)
Zn2^i^—Eu1—As5^ii^	107.39 (4)	As1^xi^—Eu6—Zn3^viii^	43.67 (2)
Zn2^ii^—Eu1—As5^ii^	51.79 (4)	As1—Eu6—Zn3^viii^	136.33 (2)
As4^i^—Eu1—As5^i^	86.40 (3)	As4^vii^—Eu6—Zn3^viii^	101.31 (4)
As4^ii^—Eu1—As5^i^	151.99 (4)	As4^iv^—Eu6—Zn3^viii^	78.69 (4)
As5^iii^—Eu1—As5^i^	102.75 (3)	As4^viii^—Eu6—Zn3^viii^	47.79 (4)
As5^iv^—Eu1—As5^i^	45.32 (4)	As4^iii^—Eu6—Zn3^viii^	132.21 (4)
Zn2^i^—Eu1—As5^i^	51.79 (4)	Zn3^vii^—Eu6—Zn3^viii^	78.21 (4)
Zn2^ii^—Eu1—As5^i^	107.39 (4)	Zn3^iv^—Eu6—Zn3^viii^	101.79 (4)
As5^ii^—Eu1—As5^i^	85.20 (4)	As1^xi^—Eu6—Zn3^iii^	136.33 (2)
As4^i^—Eu1—Zn3^ii^	105.52 (4)	As1—Eu6—Zn3^iii^	43.67 (2)
As4^ii^—Eu1—Zn3^ii^	49.41 (4)	As4^vii^—Eu6—Zn3^iii^	78.69 (4)
As5^iii^—Eu1—Zn3^ii^	61.07 (4)	As4^iv^—Eu6—Zn3^iii^	101.31 (4)
As5^iv^—Eu1—Zn3^ii^	114.41 (4)	As4^viii^—Eu6—Zn3^iii^	132.21 (4)
Zn2^i^—Eu1—Zn3^ii^	148.64 (5)	As4^iii^—Eu6—Zn3^iii^	47.79 (4)
Zn2^ii^—Eu1—Zn3^ii^	87.89 (4)	Zn3^vii^—Eu6—Zn3^iii^	101.79 (4)
As5^ii^—Eu1—Zn3^ii^	91.95 (3)	Zn3^iv^—Eu6—Zn3^iii^	78.21 (4)
As5^i^—Eu1—Zn3^ii^	157.49 (4)	Zn3^viii^—Eu6—Zn3^iii^	180.00 (8)
As4^i^—Eu1—Zn3^i^	49.41 (4)	As1^xi^—Eu6—Eu3	125.98 (3)
As4^ii^—Eu1—Zn3^i^	105.52 (4)	As1—Eu6—Eu3	54.02 (3)
As5^iii^—Eu1—Zn3^i^	114.41 (4)	As4^vii^—Eu6—Eu3	56.08 (2)
As5^iv^—Eu1—Zn3^i^	61.07 (4)	As4^iv^—Eu6—Eu3	123.92 (2)
Zn2^i^—Eu1—Zn3^i^	87.89 (4)	As4^viii^—Eu6—Eu3	56.08 (2)
Zn2^ii^—Eu1—Zn3^i^	148.64 (5)	As4^iii^—Eu6—Eu3	123.92 (2)
As5^ii^—Eu1—Zn3^i^	157.49 (4)	Zn3^vii^—Eu6—Eu3	101.40 (3)
As5^i^—Eu1—Zn3^i^	91.95 (3)	Zn3^iv^—Eu6—Eu3	78.60 (3)
Zn3^ii^—Eu1—Zn3^i^	82.18 (4)	Zn3^viii^—Eu6—Eu3	101.40 (3)
As4^i^—Eu1—As1^v^	80.45 (3)	Zn3^iii^—Eu6—Eu3	78.60 (3)
As4^ii^—Eu1—As1^v^	80.45 (3)	As1^xi^—Eu6—Eu3^xi^	54.02 (3)
As5^iii^—Eu1—As1^v^	81.93 (3)	As1—Eu6—Eu3^xi^	125.98 (3)
As5^iv^—Eu1—As1^v^	81.93 (3)	As4^vii^—Eu6—Eu3^xi^	123.92 (2)
Zn2^i^—Eu1—As1^v^	126.98 (3)	As4^iv^—Eu6—Eu3^xi^	56.08 (2)
Zn2^ii^—Eu1—As1^v^	126.98 (3)	As4^viii^—Eu6—Eu3^xi^	123.92 (2)
As5^ii^—Eu1—As1^v^	125.62 (3)	As4^iii^—Eu6—Eu3^xi^	56.08 (2)
As5^i^—Eu1—As1^v^	125.62 (3)	Zn3^vii^—Eu6—Eu3^xi^	78.60 (3)
Zn3^ii^—Eu1—As1^v^	41.63 (2)	Zn3^iv^—Eu6—Eu3^xi^	101.40 (3)
Zn3^i^—Eu1—As1^v^	41.63 (2)	Zn3^viii^—Eu6—Eu3^xi^	78.60 (3)
As4^i^—Eu1—As2	75.82 (3)	Zn3^iii^—Eu6—Eu3^xi^	101.40 (3)
As4^ii^—Eu1—As2	75.82 (3)	Eu3—Eu6—Eu3^xi^	180.0
As5^iii^—Eu1—As2	120.82 (3)	Zn3^iii^—As1—Zn3^iv^	123.85 (9)
As5^iv^—Eu1—As2	120.82 (3)	Zn3^iii^—As1—Eu2	88.04 (5)
Zn2^i^—Eu1—As2	42.68 (2)	Zn3^iv^—As1—Eu2	88.04 (5)
Zn2^ii^—Eu1—As2	42.68 (2)	Zn3^iii^—As1—Eu6	75.00 (5)
As5^ii^—Eu1—As2	76.23 (3)	Zn3^iv^—As1—Eu6	75.00 (5)
As5^i^—Eu1—As2	76.23 (3)	Eu2—As1—Eu6	142.47 (5)
Zn3^ii^—Eu1—As2	124.78 (3)	Zn3^iii^—As1—Eu3	107.52 (5)
Zn3^i^—Eu1—As2	124.78 (3)	Zn3^iv^—As1—Eu3	107.52 (5)
As1^v^—Eu1—As2	146.58 (4)	Eu2—As1—Eu3	144.42 (5)
As2—Eu2—As1	176.86 (4)	Eu6—As1—Eu3	73.11 (4)
As2—Eu2—As5^ii^	88.83 (3)	Zn3^iii^—As1—Eu4	116.04 (4)
As1—Eu2—As5^ii^	93.38 (3)	Zn3^iv^—As1—Eu4	116.04 (4)
As2—Eu2—As5^i^	88.83 (3)	Eu2—As1—Eu4	73.03 (3)
As1—Eu2—As5^i^	93.38 (3)	Eu6—As1—Eu4	144.50 (5)
As5^ii^—Eu2—As5^i^	90.31 (4)	Eu3—As1—Eu4	71.39 (3)
As2—Eu2—Zn1	57.82 (4)	Zn3^iii^—As1—Eu1^v^	63.11 (4)
As1—Eu2—Zn1	119.04 (4)	Zn3^iv^—As1—Eu1^v^	63.11 (4)
As5^ii^—Eu2—Zn1	125.86 (3)	Eu2—As1—Eu1^v^	69.76 (3)
As5^i^—Eu2—Zn1	125.86 (3)	Eu6—As1—Eu1^v^	72.71 (3)
As2—Eu2—As3^i^	84.27 (3)	Eu3—As1—Eu1^v^	145.82 (4)
As1—Eu2—As3^i^	93.44 (3)	Eu4—As1—Eu1^v^	142.79 (5)
As5^ii^—Eu2—As3^i^	172.86 (4)	Zn2^ii^—As2—Zn2^i^	117.67 (8)
As5^i^—Eu2—As3^i^	91.39 (3)	Zn2^ii^—As2—Zn1	113.26 (5)
Zn1—Eu2—As3^i^	48.25 (3)	Zn2^i^—As2—Zn1	113.26 (5)
As2—Eu2—As3^ii^	84.27 (3)	Zn2^ii^—As2—Eu2	83.18 (5)
As1—Eu2—As3^ii^	93.44 (3)	Zn2^i^—As2—Eu2	83.18 (5)
As5^ii^—Eu2—As3^ii^	91.39 (3)	Zn1—As2—Eu2	62.99 (4)
As5^i^—Eu2—As3^ii^	172.86 (4)	Zn2^ii^—As2—Eu5^i^	165.57 (6)
Zn1—Eu2—As3^ii^	48.25 (3)	Zn2^i^—As2—Eu5^i^	74.97 (4)
As3^i^—Eu2—As3^ii^	86.09 (4)	Zn1—As2—Eu5^i^	63.83 (3)
As2—Eu2—Zn2^i^	42.80 (3)	Eu2—As2—Eu5^i^	106.27 (4)
As1—Eu2—Zn2^i^	138.72 (3)	Zn2^ii^—As2—Eu5^ii^	74.97 (4)
As5^ii^—Eu2—Zn2^i^	99.04 (4)	Zn2^i^—As2—Eu5^ii^	165.57 (6)
As5^i^—Eu2—Zn2^i^	47.69 (3)	Zn1—As2—Eu5^ii^	63.83 (3)
Zn1—Eu2—Zn2^i^	84.32 (4)	Eu2—As2—Eu5^ii^	106.27 (4)
As3^i^—Eu2—Zn2^i^	77.09 (3)	Eu5^i^—As2—Eu5^ii^	91.70 (4)
As3^ii^—Eu2—Zn2^i^	125.16 (4)	Zn2^ii^—As2—Eu3^xii^	82.55 (5)
As2—Eu2—Zn2^ii^	42.80 (3)	Zn2^i^—As2—Eu3^xii^	82.55 (5)
As1—Eu2—Zn2^ii^	138.72 (3)	Zn1—As2—Eu3^xii^	144.78 (6)
As5^ii^—Eu2—Zn2^ii^	47.69 (3)	Eu2—As2—Eu3^xii^	152.23 (5)
As5^i^—Eu2—Zn2^ii^	99.04 (4)	Eu5^i^—As2—Eu3^xii^	92.82 (4)
Zn1—Eu2—Zn2^ii^	84.32 (4)	Eu5^ii^—As2—Eu3^xii^	92.82 (4)
As3^i^—Eu2—Zn2^ii^	125.16 (4)	Zn2^ii^—As2—Eu1	58.88 (4)
As3^ii^—Eu2—Zn2^ii^	77.09 (3)	Zn2^i^—As2—Eu1	58.88 (4)
Zn2^i^—Eu2—Zn2^ii^	71.68 (4)	Zn1—As2—Eu1	136.67 (6)
As2—Eu2—Eu4	121.41 (4)	Eu2—As2—Eu1	73.68 (3)
As1—Eu2—Eu4	55.45 (3)	Eu5^i^—As2—Eu1	133.69 (2)
As5^ii^—Eu2—Eu4	127.75 (3)	Eu5^ii^—As2—Eu1	133.69 (2)
As5^i^—Eu2—Eu4	127.75 (3)	Eu3^xii^—As2—Eu1	78.55 (3)
Zn1—Eu2—Eu4	63.59 (4)	Zn1^ii^—As3—Zn1^i^	114.49 (8)
As3^i^—Eu2—Eu4	55.54 (3)	Zn1^ii^—As3—Eu4	72.10 (5)
As3^ii^—Eu2—Eu4	55.54 (3)	Zn1^i^—As3—Eu4	72.10 (5)
Zn2^i^—Eu2—Eu4	132.58 (3)	Zn1^ii^—As3—Eu2^i^	136.45 (6)
Zn2^ii^—Eu2—Eu4	132.58 (3)	Zn1^i^—As3—Eu2^i^	64.95 (4)
As2—Eu2—Eu1^v^	121.40 (3)	Eu4—As3—Eu2^i^	135.58 (2)
As1—Eu2—Eu1^v^	61.74 (3)	Zn1^ii^—As3—Eu2^ii^	64.95 (4)
As5^ii^—Eu2—Eu1^v^	53.77 (3)	Zn1^i^—As3—Eu2^ii^	136.45 (6)
As5^i^—Eu2—Eu1^v^	53.77 (3)	Eu4—As3—Eu2^ii^	135.58 (2)
Zn1—Eu2—Eu1^v^	179.22 (4)	Eu2^i^—As3—Eu2^ii^	86.09 (4)
As3^i^—Eu2—Eu1^v^	132.06 (2)	Zn1^ii^—As3—Eu4^i^	152.71 (6)
As3^ii^—Eu2—Eu1^v^	132.06 (2)	Zn1^i^—As3—Eu4^i^	76.44 (4)
Zn2^i^—Eu2—Eu1^v^	95.05 (3)	Eu4—As3—Eu4^i^	89.20 (3)
Zn2^ii^—Eu2—Eu1^v^	95.05 (3)	Eu2^i^—As3—Eu4^i^	70.69 (2)
Eu4—Eu2—Eu1^v^	117.19 (3)	Eu2^ii^—As3—Eu4^i^	125.51 (5)
As2—Eu2—Eu1	60.49 (3)	Zn1^ii^—As3—Eu4^ii^	76.44 (4)
As1—Eu2—Eu1	122.65 (3)	Zn1^i^—As3—Eu4^ii^	152.71 (6)
As5^ii^—Eu2—Eu1	51.43 (2)	Eu4—As3—Eu4^ii^	89.20 (3)
As5^i^—Eu2—Eu1	51.43 (2)	Eu2^i^—As3—Eu4^ii^	125.51 (5)
Zn1—Eu2—Eu1	118.31 (4)	Eu2^ii^—As3—Eu4^ii^	70.69 (2)
As3^i^—Eu2—Eu1	125.51 (3)	Eu4^i^—As3—Eu4^ii^	83.79 (4)
As3^ii^—Eu2—Eu1	125.51 (3)	Zn1^ii^—As3—Eu5	59.56 (4)
Zn2^i^—Eu2—Eu1	48.59 (2)	Zn1^i^—As3—Eu5	59.56 (4)
Zn2^ii^—Eu2—Eu1	48.59 (2)	Eu4—As3—Eu5	75.93 (3)
Eu4—Eu2—Eu1	178.10 (2)	Eu2^i^—As3—Eu5	91.36 (3)
Eu1^v^—Eu2—Eu1	60.91 (3)	Eu2^ii^—As3—Eu5	91.36 (3)
As2^vi^—Eu3—As1	169.05 (4)	Eu4^i^—As3—Eu5	135.93 (2)
As2^vi^—Eu3—As6^iv^	93.68 (3)	Eu4^ii^—As3—Eu5	135.93 (2)
As1—Eu3—As6^iv^	94.47 (4)	Zn2—As4—Zn3	118.37 (7)
As2^vi^—Eu3—As6^iii^	93.68 (3)	Zn2—As4—Eu1^i^	67.91 (4)
As1—Eu3—As6^iii^	94.47 (4)	Zn3—As4—Eu1^i^	69.09 (4)
As6^iv^—Eu3—As6^iii^	83.84 (4)	Zn2—As4—Eu1^ii^	67.91 (4)
As2^vi^—Eu3—As4^vii^	82.40 (3)	Zn3—As4—Eu1^ii^	69.09 (4)
As1—Eu3—As4^vii^	89.39 (4)	Eu1^i^—As4—Eu1^ii^	88.62 (4)
As6^iv^—Eu3—As4^vii^	176.07 (4)	Zn2—As4—Eu6^xiii^	136.452 (19)
As6^iii^—Eu3—As4^vii^	96.65 (3)	Zn3—As4—Eu6^xiii^	71.71 (4)
As2^vi^—Eu3—As4^viii^	82.40 (3)	Eu1^i^—As4—Eu6^xiii^	140.75 (5)
As1—Eu3—As4^viii^	89.39 (4)	Eu1^ii^—As4—Eu6^xiii^	79.23 (2)
As6^iv^—Eu3—As4^viii^	96.65 (3)	Zn2—As4—Eu6^xiv^	136.452 (19)
As6^iii^—Eu3—As4^viii^	176.07 (4)	Zn3—As4—Eu6^xiv^	71.71 (4)
As4^vii^—Eu3—As4^viii^	82.59 (4)	Eu1^i^—As4—Eu6^xiv^	79.23 (2)
As2^vi^—Eu3—Zn2^vii^	42.65 (3)	Eu1^ii^—As4—Eu6^xiv^	140.75 (5)
As1—Eu3—Zn2^vii^	131.07 (3)	Eu6^xiii^—As4—Eu6^xiv^	86.96 (4)
As6^iv^—Eu3—Zn2^vii^	133.76 (4)	Zn2—As4—Eu3^vii^	77.53 (4)
As6^iii^—Eu3—Zn2^vii^	84.86 (3)	Zn3—As4—Eu3^vii^	137.37 (2)
As4^vii^—Eu3—Zn2^vii^	42.58 (3)	Eu1^i^—As4—Eu3^vii^	144.88 (5)
As4^viii^—Eu3—Zn2^vii^	92.03 (3)	Eu1^ii^—As4—Eu3^vii^	84.03 (2)
As2^vi^—Eu3—Zn2^viii^	42.65 (3)	Eu6^xiii^—As4—Eu3^vii^	71.18 (3)
As1—Eu3—Zn2^viii^	131.07 (3)	Eu6^xiv^—As4—Eu3^vii^	125.84 (5)
As6^iv^—Eu3—Zn2^viii^	84.86 (3)	Zn2—As4—Eu3^viii^	77.53 (4)
As6^iii^—Eu3—Zn2^viii^	133.76 (4)	Zn3—As4—Eu3^viii^	137.37 (2)
As4^vii^—Eu3—Zn2^viii^	92.03 (3)	Eu1^i^—As4—Eu3^viii^	84.03 (2)
As4^viii^—Eu3—Zn2^viii^	42.58 (3)	Eu1^ii^—As4—Eu3^viii^	144.88 (5)
Zn2^vii^—Eu3—Zn2^viii^	71.56 (4)	Eu6^xiii^—As4—Eu3^viii^	125.84 (5)
As2^vi^—Eu3—Eu4	136.26 (4)	Eu6^xiv^—As4—Eu3^viii^	71.18 (3)
As1—Eu3—Eu4	54.69 (3)	Eu3^vii^—As4—Eu3^viii^	82.59 (4)
As6^iv^—Eu3—Eu4	56.02 (2)	As5^xv^—As5—Zn2	111.14 (8)
As6^iii^—Eu3—Eu4	56.02 (2)	As5^xv^—As5—Eu2^ii^	134.67 (2)
As4^vii^—Eu3—Eu4	127.36 (3)	Zn2—As5—Eu2^ii^	78.34 (4)
As4^viii^—Eu3—Eu4	127.36 (3)	As5^xv^—As5—Eu2^i^	134.67 (2)
Zn2^vii^—Eu3—Eu4	140.18 (2)	Zn2—As5—Eu2^i^	78.34 (4)
Zn2^viii^—Eu3—Eu4	140.18 (2)	Eu2^ii^—As5—Eu2^i^	90.31 (4)
As2^vi^—Eu3—Eu6	116.17 (3)	As5^xv^—As5—Eu1^xiv^	67.89 (4)
As1—Eu3—Eu6	52.87 (3)	Zn2—As5—Eu1^xiv^	136.08 (2)
As6^iv^—Eu3—Eu6	129.57 (2)	Eu2^ii^—As5—Eu1^xiv^	135.55 (5)
As6^iii^—Eu3—Eu6	129.57 (3)	Eu2^i^—As5—Eu1^xiv^	75.31 (3)
As4^vii^—Eu3—Eu6	52.74 (2)	As5^xv^—As5—Eu1^xiii^	67.89 (4)
As4^viii^—Eu3—Eu6	52.74 (2)	Zn2—As5—Eu1^xiii^	136.08 (2)
Zn2^vii^—Eu3—Eu6	90.98 (3)	Eu2^ii^—As5—Eu1^xiii^	75.31 (3)
Zn2^viii^—Eu3—Eu6	90.98 (3)	Eu2^i^—As5—Eu1^xiii^	135.55 (5)
Eu4—Eu3—Eu6	107.57 (2)	Eu1^xiv^—As5—Eu1^xiii^	86.05 (4)
As2^vi^—Eu3—Eu1^vi^	57.02 (3)	As5^xv^—As5—Eu1^ii^	66.79 (4)
As1—Eu3—Eu1^vi^	112.03 (4)	Zn2—As5—Eu1^ii^	64.04 (4)
As6^iv^—Eu3—Eu1^vi^	131.05 (2)	Eu2^ii^—As5—Eu1^ii^	80.29 (2)
As6^iii^—Eu3—Eu1^vi^	131.05 (2)	Eu2^i^—As5—Eu1^ii^	142.30 (5)
As4^vii^—Eu3—Eu1^vi^	46.18 (2)	Eu1^xiv^—As5—Eu1^ii^	134.68 (4)
As4^viii^—Eu3—Eu1^vi^	46.18 (2)	Eu1^xiii^—As5—Eu1^ii^	77.25 (3)
Zn2^vii^—Eu3—Eu1^vi^	46.56 (2)	As5^xv^—As5—Eu1^i^	66.79 (4)
Zn2^viii^—Eu3—Eu1^vi^	46.56 (2)	Zn2—As5—Eu1^i^	64.04 (4)
Eu4—Eu3—Eu1^vi^	166.72 (2)	Eu2^ii^—As5—Eu1^i^	142.30 (5)
Eu6—Eu3—Eu1^vi^	59.155 (18)	Eu2^i^—As5—Eu1^i^	80.29 (2)
As2^vi^—Eu3—Eu3^ix^	90.0	Eu1^xiv^—As5—Eu1^i^	77.25 (3)
As1—Eu3—Eu3^ix^	90.0	Eu1^xiii^—As5—Eu1^i^	134.68 (4)
As6^iv^—Eu3—Eu3^ix^	48.08 (2)	Eu1^ii^—As5—Eu1^i^	85.21 (4)
As6^iii^—Eu3—Eu3^ix^	131.919 (19)	Zn1^xv^—As6—Eu5^xiv^	66.73 (4)
As4^vii^—Eu3—Eu3^ix^	131.297 (19)	Zn1^xv^—As6—Eu5^xiii^	66.73 (4)
As4^viii^—Eu3—Eu3^ix^	48.705 (19)	Eu5^xiv^—As6—Eu5^xiii^	86.72 (4)
Zn2^vii^—Eu3—Eu3^ix^	125.780 (18)	Zn1^xv^—As6—Eu3^xiii^	134.69 (3)
Zn2^viii^—Eu3—Eu3^ix^	54.221 (18)	Eu5^xiv^—As6—Eu3^xiii^	152.24 (5)
Eu4—Eu3—Eu3^ix^	90.0	Eu5^xiii^—As6—Eu3^xiii^	88.14 (3)
Eu6—Eu3—Eu3^ix^	90.001 (1)	Zn1^xv^—As6—Eu3^xiv^	134.69 (3)
Eu1^vi^—Eu3—Eu3^ix^	90.0	Eu5^xiv^—As6—Eu3^xiv^	88.14 (3)
As3—Eu4—As1	179.95 (4)	Eu5^xiii^—As6—Eu3^xiv^	152.24 (5)
As3—Eu4—As3^i^	90.81 (3)	Eu3^xiii^—As6—Eu3^xiv^	83.84 (4)
As1—Eu4—As3^i^	89.23 (3)	Zn1^xv^—As6—Eu4^xiv^	69.45 (4)
As3—Eu4—As3^ii^	90.81 (3)	Eu5^xiv^—As6—Eu4^xiv^	79.41 (2)
As1—Eu4—As3^ii^	89.23 (3)	Eu5^xiii^—As6—Eu4^xiv^	136.02 (5)
As3^i^—Eu4—As3^ii^	83.79 (4)	Eu3^xiii^—As6—Eu4^xiv^	121.64 (4)
As3—Eu4—As6^iii^	87.04 (3)	Eu3^xiv^—As6—Eu4^xiv^	69.23 (3)
As1—Eu4—As6^iii^	92.92 (3)	Zn1^xv^—As6—Eu4^xiii^	69.45 (4)
As3^i^—Eu4—As6^iii^	177.74 (4)	Eu5^xiv^—As6—Eu4^xiii^	136.02 (5)
As3^ii^—Eu4—As6^iii^	96.93 (3)	Eu5^xiii^—As6—Eu4^xiii^	79.41 (2)
As3—Eu4—As6^iv^	87.04 (3)	Eu3^xiii^—As6—Eu4^xiii^	69.23 (3)
As1—Eu4—As6^iv^	92.92 (3)	Eu3^xiv^—As6—Eu4^xiii^	121.64 (4)
As3^i^—Eu4—As6^iv^	96.93 (3)	Eu4^xiv^—As6—Eu4^xiii^	82.28 (4)
As3^ii^—Eu4—As6^iv^	177.74 (4)	Zn1^xv^—As6—Eu5^x^	119.13 (6)
As6^iii^—Eu4—As6^iv^	82.28 (4)	Eu5^xiv^—As6—Eu5^x^	70.08 (3)
As3—Eu4—Zn1^i^	46.61 (3)	Eu5^xiii^—As6—Eu5^x^	70.08 (3)
As1—Eu4—Zn1^i^	133.37 (3)	Eu3^xiii^—As6—Eu5^x^	82.52 (3)
As3^i^—Eu4—Zn1^i^	80.44 (3)	Eu3^xiv^—As6—Eu5^x^	82.52 (3)
As3^ii^—Eu4—Zn1^i^	133.75 (4)	Eu4^xiv^—As6—Eu5^x^	138.84 (2)
As6^iii^—Eu4—Zn1^i^	97.56 (4)	Eu4^xiii^—As6—Eu5^x^	138.844 (19)
As6^iv^—Eu4—Zn1^i^	44.49 (3)	As6^xv^—Zn1—As3^ii^	119.71 (4)
As3—Eu4—Zn1^ii^	46.61 (3)	As6^xv^—Zn1—As3^i^	119.71 (4)
As1—Eu4—Zn1^ii^	133.37 (3)	As3^ii^—Zn1—As3^i^	114.49 (8)
As3^i^—Eu4—Zn1^ii^	133.75 (4)	As6^xv^—Zn1—As2	101.21 (7)
As3^ii^—Eu4—Zn1^ii^	80.44 (3)	As3^ii^—Zn1—As2	96.70 (5)
As6^iii^—Eu4—Zn1^ii^	44.49 (3)	As3^i^—Zn1—As2	96.70 (5)
As6^iv^—Eu4—Zn1^ii^	97.56 (4)	As6^xv^—Zn1—Eu2	160.40 (7)
Zn1^i^—Eu4—Zn1^ii^	79.91 (4)	As3^ii^—Zn1—Eu2	66.80 (4)
As3—Eu4—Zn1	78.17 (4)	As3^i^—Zn1—Eu2	66.80 (4)
As1—Eu4—Zn1	101.88 (4)	As2—Zn1—Eu2	59.19 (5)
As3^i^—Eu4—Zn1	43.49 (2)	As6^xv^—Zn1—Eu5^i^	66.21 (4)
As3^ii^—Eu4—Zn1	43.49 (2)	As3^ii^—Zn1—Eu5^i^	155.11 (7)
As6^iii^—Eu4—Zn1	136.45 (2)	As3^i^—Zn1—Eu5^i^	75.93 (4)
As6^iv^—Eu4—Zn1	136.45 (2)	As2—Zn1—Eu5^i^	58.81 (3)
Zn1^i^—Eu4—Zn1	101.07 (4)	Eu2—Zn1—Eu5^i^	100.14 (4)
Zn1^ii^—Eu4—Zn1	101.07 (4)	As6^xv^—Zn1—Eu5^ii^	66.21 (4)
As3—Eu4—Eu3	126.04 (3)	As3^ii^—Zn1—Eu5^ii^	75.93 (4)
As1—Eu4—Eu3	53.92 (3)	As3^i^—Zn1—Eu5^ii^	155.11 (7)
As3^i^—Eu4—Eu3	126.41 (3)	As2—Zn1—Eu5^ii^	58.81 (3)
As3^ii^—Eu4—Eu3	126.41 (3)	Eu2—Zn1—Eu5^ii^	100.14 (4)
As6^iii^—Eu4—Eu3	54.74 (3)	Eu5^i^—Zn1—Eu5^ii^	86.29 (5)
As6^iv^—Eu4—Eu3	54.74 (3)	As6^xv^—Zn1—Eu4^i^	66.06 (4)
Zn1^i^—Eu4—Eu3	97.42 (3)	As3^ii^—Zn1—Eu4^i^	126.86 (7)
Zn1^ii^—Eu4—Eu3	97.42 (3)	As3^i^—Zn1—Eu4^i^	61.30 (4)
Zn1—Eu4—Eu3	155.80 (3)	As2—Zn1—Eu4^i^	135.80 (3)
As3—Eu4—Eu2	128.52 (4)	Eu2—Zn1—Eu4^i^	126.90 (4)
As1—Eu4—Eu2	51.53 (3)	Eu5^i^—Zn1—Eu4^i^	78.03 (3)
As3^i^—Eu4—Eu2	53.77 (2)	Eu5^ii^—Zn1—Eu4^i^	132.14 (5)
As3^ii^—Eu4—Eu2	53.77 (2)	As6^xv^—Zn1—Eu4^ii^	66.06 (4)
As6^iii^—Eu4—Eu2	128.31 (3)	As3^ii^—Zn1—Eu4^ii^	61.30 (4)
As6^iv^—Eu4—Eu2	128.31 (3)	As3^i^—Zn1—Eu4^ii^	126.86 (7)
Zn1^i^—Eu4—Eu2	133.94 (3)	As2—Zn1—Eu4^ii^	135.80 (3)
Zn1^ii^—Eu4—Eu2	133.94 (3)	Eu2—Zn1—Eu4^ii^	126.90 (4)
Zn1—Eu4—Eu2	50.35 (3)	Eu5^i^—Zn1—Eu4^ii^	132.14 (5)
Eu3—Eu4—Eu2	105.44 (3)	Eu5^ii^—Zn1—Eu4^ii^	78.03 (3)
As3—Eu4—Eu5	57.03 (3)	Eu4^i^—Zn1—Eu4^ii^	79.91 (4)
As1—Eu4—Eu5	122.92 (3)	As6^xv^—Zn1—Eu4	133.54 (7)
As3^i^—Eu4—Eu5	129.20 (3)	As3^ii^—Zn1—Eu4	60.08 (4)
As3^ii^—Eu4—Eu5	129.20 (3)	As3^i^—Zn1—Eu4	60.08 (4)
As6^iii^—Eu4—Eu5	48.82 (2)	As2—Zn1—Eu4	125.24 (6)
As6^iv^—Eu4—Eu5	48.82 (2)	Eu2—Zn1—Eu4	66.06 (3)
Zn1^i^—Eu4—Eu5	48.77 (3)	Eu5^i^—Zn1—Eu4	135.94 (3)
Zn1^ii^—Eu4—Eu5	48.77 (3)	Eu5^ii^—Zn1—Eu4	135.94 (3)
Zn1—Eu4—Eu5	135.20 (3)	Eu4^i^—Zn1—Eu4	78.93 (4)
Eu3—Eu4—Eu5	69.01 (3)	Eu4^ii^—Zn1—Eu4	78.93 (4)
Eu2—Eu4—Eu5	174.45 (2)	As2^ii^—Zn2—As2^i^	117.66 (8)
As2^i^—Eu5—As2^ii^	91.69 (4)	As2^ii^—Zn2—As4	109.99 (5)
As2^i^—Eu5—As6^iii^	159.31 (4)	As2^i^—Zn2—As4	109.99 (5)
As2^ii^—Eu5—As6^iii^	87.15 (3)	As2^ii^—Zn2—As5	105.73 (5)
As2^i^—Eu5—As6^iv^	87.15 (3)	As2^i^—Zn2—As5	105.73 (5)
As2^ii^—Eu5—As6^iv^	159.31 (4)	As4—Zn2—As5	107.07 (7)
As6^iii^—Eu5—As6^iv^	86.72 (4)	As2^ii^—Zn2—Eu1^i^	163.56 (6)
As2^i^—Eu5—Zn1^i^	57.36 (4)	As2^i^—Zn2—Eu1^i^	78.43 (3)
As2^ii^—Eu5—Zn1^i^	116.22 (4)	As4—Zn2—Eu1^i^	64.00 (4)
As6^iii^—Eu5—Zn1^i^	104.95 (4)	As5—Zn2—Eu1^i^	64.16 (4)
As6^iv^—Eu5—Zn1^i^	47.06 (4)	As2^ii^—Zn2—Eu1^ii^	78.43 (3)
As2^i^—Eu5—Zn1^ii^	116.22 (4)	As2^i^—Zn2—Eu1i	163.56 (6)
As2^ii^—Eu5—Zn1^ii^	57.36 (4)	As4—Zn2—Eu1i	64.00 (4)
As6^iii^—Eu5—Zn1^ii^	47.06 (4)	As5—Zn2—Eu1i	64.16 (4)
As6^iv^—Eu5—Zn1^ii^	104.95 (4)	Eu1i—Zn2—Eu1i	85.31 (4)
Zn1^i^—Eu5—Zn1^ii^	86.29 (5)	As2i—Zn2—Eu5	59.05 (4)
As2^i^—Eu5—Zn2	45.98 (2)	As2i—Zn2—Eu5	59.05 (4)
As2^ii^—Eu5—Zn2	45.98 (2)	As4—Zn2—Eu5	124.90 (7)
As6^iii^—Eu5—Zn2	131.21 (3)	As5—Zn2—Eu5	128.03 (6)
As6^iv^—Eu5—Zn2	131.21 (3)	Eu1i—Zn2—Eu5	137.32 (2)
Zn1^i^—Eu5—Zn2	88.91 (4)	Eu1i—Zn2—Eu5	137.32 (2)
Zn1^ii^—Eu5—Zn2	88.91 (4)	As2i—Zn2—Eu2i	114.49 (6)
As2^i^—Eu5—As6^x^	90.74 (3)	As2i—Zn2—Eu2i	54.02 (4)
As2^ii^—Eu5—As6^x^	90.74 (3)	As4—Zn2—Eu2i	134.91 (4)
As6^iii^—Eu5—As6^x^	109.93 (3)	As5—Zn2—Eu2i	53.97 (3)
As6^iv^—Eu5—As6^x^	109.93 (3)	Eu1i—Zn2—Eu2i	71.18 (3)
Zn1^i^—Eu5—As6^x^	136.67 (2)	Eu1i—Zn2—Eu2i	118.09 (5)
Zn1^ii^—Eu5—As6^x^	136.67 (2)	Eu5—Zn2—Eu2i	85.58 (3)
Zn2—Eu5—As6^x^	87.06 (4)	As2i—Zn2—Eu2i	54.02 (4)
As2^i^—Eu5—As3	77.76 (3)	As2i—Zn2—Eu2i	114.49 (6)
As2^ii^—Eu5—As3	77.76 (3)	As4—Zn2—Eu2i	134.91 (4)
As6^iii^—Eu5—As3	81.81 (3)	As5—Zn2—Eu2i	53.97 (3)
As6^iv^—Eu5—As3	81.81 (3)	Eu1i—Zn2—Eu2i	118.09 (5)
Zn1^i^—Eu5—As3	44.51 (2)	Eu1i—Zn2—Eu2i	71.18 (3)
Zn1^ii^—Eu5—As3	44.51 (2)	Eu5—Zn2—Eu2i	85.58 (3)
Zn2—Eu5—As3	76.28 (4)	Eu2i—Zn2—Eu2i	71.68 (4)
As6^x^—Eu5—As3	163.35 (4)	As2i—Zn2—Eu3i	54.79 (4)
As2^i^—Eu5—Eu5^vii^	142.36 (4)	As2i—Zn2—Eu3i	115.09 (6)
As2^ii^—Eu5—Eu5^vii^	88.30 (3)	As4—Zn2—Eu3i	59.89 (4)
As6^iii^—Eu5—Eu5^vii^	58.29 (3)	As5—Zn2—Eu3i	139.18 (3)
As6^iv^—Eu5—Eu5^vii^	105.11 (4)	Eu1i—Zn2—Eu3i	123.56 (5)
Zn1^i^—Eu5—Eu5^vii^	150.85 (4)	Eu1i—Zn2—Eu3i	76.14 (3)
Zn1^ii^—Eu5—Eu5^vii^	95.17 (3)	Eu5—Zn2—Eu3i	76.36 (3)
Zn2—Eu5—Eu5^vii^	120.20 (3)	Eu2i—Zn2—Eu3i	161.91 (5)
As6^x^—Eu5—Eu5^vii^	51.63 (3)	Eu2i—Zn2—Eu3i	105.42 (2)
As3—Eu5—Eu5^vii^	138.43 (2)	As2i—Zn2—Eu3i	115.09 (6)
As2^i^—Eu5—Eu5^viii^	88.30 (3)	As2i—Zn2—Eu3i	54.79 (4)
As2^ii^—Eu5—Eu5^viii^	142.36 (4)	As4—Zn2—Eu3i	59.89 (4)
As6^iii^—Eu5—Eu5^viii^	105.11 (4)	As5—Zn2—Eu3i	139.18 (3)
As6^iv^—Eu5—Eu5^viii^	58.29 (3)	Eu1i—Zn2—Eu3i	76.14 (3)
Zn1^i^—Eu5—Eu5^viii^	95.17 (3)	Eu1i—Zn2—Eu3i	123.56 (5)
Zn1^ii^—Eu5—Eu5^viii^	150.85 (4)	Eu5—Zn2—Eu3i	76.36 (3)
Zn2—Eu5—Eu5^viii^	120.20 (3)	Eu2i—Zn2—Eu3i	105.42 (2)
As6^x^—Eu5—Eu5^viii^	51.63 (3)	Eu2i—Zn2—Eu3i	161.91 (5)
As3—Eu5—Eu5^viii^	138.43 (2)	Eu3i—Zn2—Eu3i	71.56 (4)
Eu5^vii^—Eu5—Eu5^viii^	69.86 (3)	As1i—Zn3—As1i	123.84 (9)
As2^i^—Eu5—Eu4	109.95 (3)	As1i—Zn3—As4	114.73 (5)
As2^ii^—Eu5—Eu4	109.95 (3)	As1i—Zn3—As4	114.73 (5)
As6^iii^—Eu5—Eu4	51.77 (2)	As1i—Zn3—Eu1i	154.86 (7)
As6^iv^—Eu5—Eu4	51.77 (2)	As1i—Zn3—Eu1i	75.26 (4)
Zn1^i^—Eu5—Eu4	53.21 (3)	As4—Zn3—Eu1i	61.51 (4)
Zn1^ii^—Eu5—Eu4	53.21 (3)	As1i—Zn3—Eu1i	75.26 (4)
Zn2—Eu5—Eu4	123.33 (4)	As1i—Zn3—Eu1i	154.86 (7)
As6^x^—Eu5—Eu4	149.61 (3)	As4—Zn3—Eu1i	61.51 (4)
As3—Eu5—Eu4	47.04 (3)	Eu1i—Zn3—Eu1i	82.18 (4)
Eu5^vii^—Eu5—Eu4	105.33 (3)	As1i—Zn3—Eu6i	129.29 (7)
Eu5^viii^—Eu5—Eu4	105.33 (3)	As1i—Zn3—Eu6i	61.33 (4)
As1^xi^—Eu6—As1	180.0	As4—Zn3—Eu6i	60.50 (3)
As1^xi^—Eu6—As4^vii^	87.28 (3)	Eu1i—Zn3—Eu6i	72.57 (3)
As1—Eu6—As4^vii^	92.72 (3)	Eu1i—Zn3—Eu6i	121.98 (5)
As1^xi^—Eu6—As4^iv^	92.72 (3)	As1i—Zn3—Eu6i	61.33 (4)
As1—Eu6—As4^iv^	87.28 (3)	As1i—Zn3—Eu6i	129.29 (7)
As4^vii^—Eu6—As4^iv^	180.00 (5)	As4—Zn3—Eu6i	60.50 (3)
As1^xi^—Eu6—As4^viii^	87.28 (3)	Eu1i—Zn3—Eu6i	121.98 (5)
As1—Eu6—As4^viii^	92.72 (3)	Eu1i—Zn3—Eu6i	72.57 (3)
As4^vii^—Eu6—As4^viii^	86.96 (4)	Eu6i—Zn3—Eu6i	78.21 (4)

Symmetry codes: (i) −*x*+1/2, −*y*+1/2, −*z*+1; (ii) −*x*+1/2, −*y*−1/2, −*z*+1; (iii) *x*−1/2, *y*−1/2, *z*; (iv) *x*−1/2, *y*+1/2, *z*; (v) −*x*, −*y*, −*z*+1; (vi) *x*, *y*, *z*−1; (vii) −*x*+1/2, −*y*−1/2, −*z*; (viii) −*x*+1/2, −*y*+1/2, −*z*; (ix) *x*, *y*+1, *z*; (x) −*x*+1, −*y*, −*z*; (xi) −*x*, −*y*, −*z*; (xii) *x*, *y*, *z*+1; (xiii) *x*+1/2, *y*−1/2, *z*; (xiv) *x*+1/2, *y*+1/2, *z*; (xv) −*x*+1, −*y*, −*z*+1.
